# Food avoidance in anorexia nervosa: associated and predicting factors

**DOI:** 10.1007/s40519-023-01545-4

**Published:** 2023-02-23

**Authors:** L. Di Lodovico, C. Vansteene, D. Poupon, P. Gorwood, P. Duriez, the FFAB, Nathalie Godart, Sébastien Guillaume, Sylvain Lambert, F. Chevallier-Latreuille, Brigitte Remy, Q. Barrois, M. Delorme, Catherine Massoubre, Vincent Dodin, Guillaume Lavoisy, Sophie Criquillion, Sylvan Iceta, C. Fayollet, Philippe Nubukpo, Florat Bat

**Affiliations:** 1 GHU Paris Psychiatry and Neurosciences, Clinic of Mental Illnesses and Brain Disorders, 75014 Paris, France; 2 Institute of Psychiatry and Neuroscience of Paris (IPNP), INSERM U1266, University of Paris, 75014 Paris, France; 3 CMME, GHU Paris Psychiatry and Neurosciences, 100 Rue de La Santé, 75674 Paris Cedex 14, France

**Keywords:** Anorexia nervosa, Food avoidance, Eating disorders, Depression, Anxiety

## Abstract

**Purpose:**

Qualitative food avoidance is a significant issue in patients with anorexia nervosa (AN) and restoring diet diversity is an important part of the treatment process. We aimed to identify clinical factors which drive food avoidance and predict its maintenance in patients with AN.

**Methods:**

In this multicentre longitudinal study, 130 female outpatients with AN were assessed before and after 4 months of care in clinical centres specialized in AN. We assessed levels of avoidance of 16 food items, as well as body mass index (BMI), eating disorder severity, symptoms of depression and anxiety, emotional state, daily-life functioning, and body image perception.

**Results:**

We found that qualitative food avoidance was associated with the clinical severity of AN, anxiety and mood dimensions, and BMI- and body image-related factors. A younger age at onset predicted the maintenance of food avoidance after 4 months of treatment. Additional exploratory analyses suggested that anxiety and negative affect caused food avoidance more than the opposite.

**Conclusion:**

Qualitative food avoidance can be an indicator of illness severity. During treatment, focusing on reducing anxiety and negative affect may be a way to indirectly reduce food avoidance and restore diet diversity.

**Level of evidence:**

Level III: Evidence obtained from cohort or case-control analytic studies.

**Supplementary Information:**

The online version contains supplementary material available at 10.1007/s40519-023-01545-4.

## Introduction

Anorexia nervosa (AN) is the eating disorder (ED) with the highest mortality rate [1]. It is a complex psychiatric disorder characterized by altered eating-related behaviours and body image distortions [2].

Patients with AN typically limit or avoid food intake [3]. High-calorie foods are their main target: patients rate their desire to eat high-calorie foods significantly lower than low-calorie foods, while no difference is observed in healthy controls [4]. Patients with AN, either restrictive (AN-R) or binge-eating/purging (AN-BP) type, tend to choose low-fat foods over high-fat foods, and to undervalue the tastiness of high-fat foods [5]. Fat intake correlates with self-reported preference for high-fat food, and both are lower in patients with AN than in healthy controls [6]. Underlying processes are involved in food avoidance, altering responses to food. For example, an eye-tracking study highlighted that patients with AN avoid maintaining attention on food cues, which potentially facilitates restrictive eating [7]. These underlying processes seem to persist in weight-restored patients. Their *explicit*, self-reported, desire to eat high-calorie foods was indeed higher than in currently underweight patients and similar to that of healthy controls, but their *implicit* desire to eat high-calorie foods (assessed through reaction times) was as low as in currently underweight patients [8].

Nutritional rehabilitation is a key element in the treatment of AN [9]. As a result of their resistance to eating a variety of foods, nutrient needs are not met in patients with AN [10, 11]. This is why, while increasing food intake is important to restore weight, increasing diversity in food selections is also essential to restore nutritional status [9]. This is especially important in the long term as diet diversity is predictive of weight maintenance [12]. However, the literature on this topic is scarce [9]. While food avoidance is a main issue in AN and nutritional rehabilitation a main challenge during treatment, there has been limited research on factors driving food avoidance and predicting its variability in patients being treated for AN.

In the present prospective longitudinal exploratory study, we examined clinical characteristics and self-rated avoidance of 16 food items before and after 4 months of treatment. Our hypothesis was that food avoidance correlates with clinical severity of AN and other factors. As the avoided high-calorie foods may vary from one patient to another, we used a principal component analysis (PCA) to homogenize the results, expecting a single component to represent avoidance of high-calorie foods. Then, we tested our hypothesis through three aspects: (1) direct association at baseline between food avoidance, clinical severity of AN and potential associated factors; (2) colinear evolution over time; (3) capacity of clinical severity score and other factors to predict food avoidance maintenance versus successful food reintroduction. Identifying associated factors could help tackle food avoidance more efficiently during treatment.

## Methods

### Participants

Female outpatients with AN were screened for inclusion in 13 centres specialized in ED throughout France, as described in more detail elsewhere [13, 14]. Recruitment took place from February 2015 to July 2016. All patients were assessed during a face‐to‐face interview with a psychiatrist (who had at least 5 years of experience in ED) and were included when fulfilling the DSM5 criteria for AN [2]. Exclusion criteria were: not being affiliated to a social security system, not being fluent in French, being illiterate, not knowing how to use a computer, or presenting with dementia or delirium. Initially, 221 outpatients were included. Twenty-one patients were excluded because mandatory clinical data were missing, and 70 were lost to follow‐up (35%). A total of 130 outpatients were therefore included in the present analyses.

Patients were assessed at admission (T1) and approximately 4 months later (T2). The average time period between first and second evaluations was 132 days (SD = 97.9). To address this variability, the delay between visits was included in the analyses.

Patient care can vary from one centre to another, but it consistently includes a multidisciplinary approach involving both a psychiatrist and/or a psychologist and a nutritionist or a dietician. All patients are offered at least one evidence-based psychotherapy for ED (cognitive–behavioural therapy, interpersonal therapy, family therapy, multifamily therapy), and psychotropic drugs are prescribed when needed (primarily antidepressants).

Participants who did not attend the follow‐up visit had a centre effect (χ^2^ = 29.257, df = 12, *p* = 0.004), and were characterized by a higher initial (16.128, SD = 2.966; *F* = 5.116, *p* = 0.025), minimum (13.919, SD = 2.159; *F* = 8.397, *p* = 0.004), and maximum (21.970, SD = 5.666; *F* = 4.441, *p* = 0.036) BMI, and lower positive (26.05, SD = 7.986; *F* = 6.534, *p* = 0.011) and negative (26.04, SD = 6.641; *F* = 55.982, *p* < 0.001) affect. Other variables did not differ [13, 14].

### Instruments

Clinical assessment included questions about current, subjective ideal, and minimum and maximum lifetime (since puberty, if present) body mass index (BMI), age at onset of AN, educational level, working activity, and the presence of a familial history of this disorder. For educational level, working activity and familial history of ED, to simplify comparisons, we divided patients into groups, i.e. university graduates versus below, working full or half-time versus not, and having at least one relative at the first or second degree diagnosed with anorexia nervosa or bulimia nervosa versus none.

Specific questionnaires and tests were provided to every patient with an established diagnosis after obtaining their consent during the first visit, at admission. All tests were repeated during the second visit, about 4 months later.

ED symptomatology was assessed using a French version of the Eating Attitudes Test-26 (EAT) [15, 16]. Three subdivisions distinguish “dieting” (13 items), “bulimia” (6 items), and “oral control” (7 items). From six-point Likert scales (from “never” to “always”), items are scored from 0 to 3 (three out of the six possible answers are rated 0). EAT total score ranges from 0 to 78. A score above 20 indicates problematic eating behaviours and a high level of concern about dieting and body weight [15].

Food avoidance was assessed through an ad hoc questionnaire constructed with a psychologist specialized in ED patients’ eating behaviours. Patients were asked to rate their levels of avoidance of 16 food items: butter, starchy foods, fries, cheese, pastries, cold meats, ham, red meat, white meat, white fish, 0% fat dairy produce, green vegetables, tomatoes, fresh fruits (except bananas), dried fruits, and whole wheat bread. Avoidance was rated on a three-point scale from 0 to 2 (0 = “I never avoid it”, 1 = “I sometimes avoid it”, 2 = “I always avoid it”).

Depression and anxiety scores were measured with the Hospital Anxiety and Depressive Scale (HADS), a self-report instrument with seven questions devoted to depression and a further seven to anxiety [17]. This instrument provides quantitative and qualitative data as, for both depression and anxiety, a score above 8 has been validated for current depressive or anxiety disorder [18]. Because the presence of a depressive or anxious disorder was evaluated both at the beginning and at the end of the protocol, we computed the number of patients in remission from these conditions during the second visit (patients with a score above 8 at T1 and a score below 8 at T2).

Patients’ emotional state was assessed using the Positive and Negative Affect Schedule (PANAS), a 10-item self-report questionnaire [19]. Each item is rated on a 5-point scale, from 1 indicating that the word does “not at all” characterize the patient, to 5 meaning it “very much” does. Both scores range from 10 to 50, with higher scores indicating higher levels of positive and negative affect, respectively.

The Work and Social Adjustment Scale (WSAS) [20] assesses the level of impairment in the ability to work, home management, to engage in social and private leisure activities, and maintain close relationships. The maximum possible score is 40, with lower scores representing better functionality.

The body image perception test was based on a diagram representing the progression of ten female silhouettes, each corresponding to a specific BMI [21]. Patients were instructed to choose the silhouette that best represented their current body. Higher scores indicate higher perceived BMI and, within the scope of this study, stronger body distortion.

### Data analysis

Statistical analyses were performed using Jamovi 1.6.23 for Windows [22] and R version 4.2.1. Significance threshold was *p* < 0.05. Normal distribution was initially checked using the Kolmogorov–Smirnov test. When variables did not have a normal distribution (*p* > 0.05), we used non-parametric tests. To limit the risk of type I errors due to multiple comparisons, we controlled the false discovery rate (FDR) using the Benjamini–Hochberg adjustment with a FDR of 5% [23, 24]. Multiple regressions were then used with variables found significant.

A principal component analysis (PCA) with varimax rotation was initially performed for dimensionality reduction. Input variables were the 16 rates of avoidance of the 16 different food items at T1. Both scree plot [25] and parallel analysis [26, 27] suggested to retain three components. Factor scores for each of the three components were computed for each patient.

To test associations between factor scores of the three components and clinical characteristics, we performed Pearson’s correlations for continuous variables and Mann–Whitney *U* tests for categorical variables.

To assess the evolution of factor scores and clinical characteristics between T1 and T2, we used Wilcoxon tests for continuous variables and Chi-squared tests for categorical variables.

We computed the categorical variable “successful food reintroduction”, defined as the presence of avoidance (avoidance ≥ 1) at T1 and the absence of avoidance (avoidance = 0) at T2 for at least one high-fat food (butter, fries, cheese, pastries, cold meats). We then compared profiles of patients with or without successful food reintroduction with Mann–Whitney *U* tests for continuous variables and with Chi-squared tests for categorical variables.

Finally, we explored the causal relationship between food avoidance and other variables with cross-lagged panel models using the R package “Lavaan”.

## Results

The final sample included 130 patients aged between 11 and 52 years old (mean age = 25.1, SD = 10.9), with a mean age at onset of 17.2 years old (SD = 4.9), an average illness duration of 7.9 years (SD = 9.5), and an average BMI of 15.3 (SD = 1.9). The sample included 74 patients (56.9%) diagnosed with AN-R type, and 56 (43.1%) with AN-BP type.

### Food avoidance at baseline

The three principal components obtained from the PCA explained, respectively, 20.1%, 15.3% and 14.5% of the variance. Component loadings indicate that the first principal component mostly reflects avoidance of high-calorie foods, the second one avoidance of animal-based foods, and the third one avoidance of fruits and vegetables (Table S1). For clarity, we refer to the first, second and third principal components as PC-caloric, PC-animal, and PC-vegetal, respectively. Higher levels of food avoidance translate into higher factor scores.

**Fig. 1:**
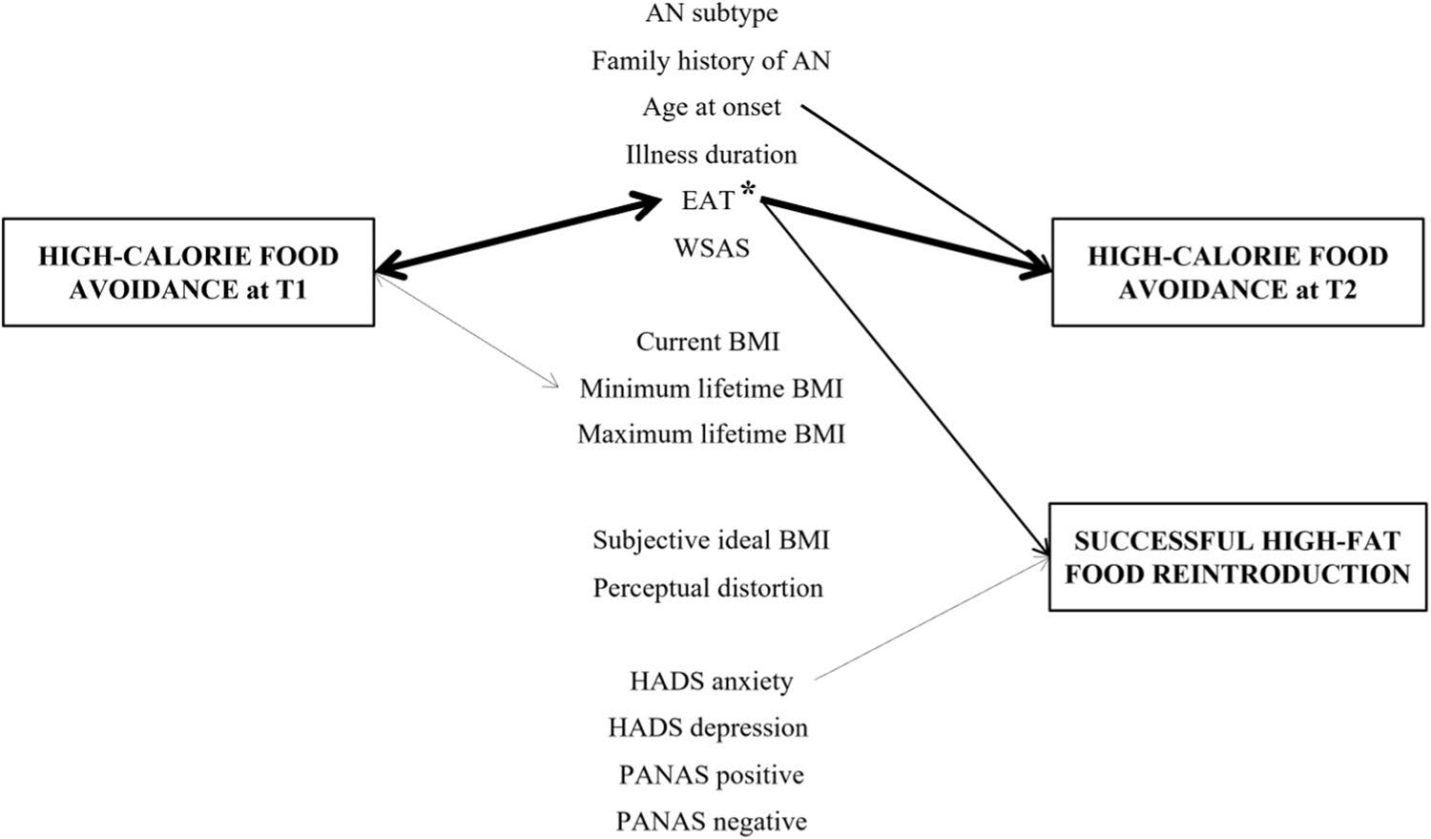
Factors associated with the description (T1) and evolution (T2) of the component “high-calorie food avoidance” according to multiple regressions. High-calorie food avoidance at baseline (T1) and after 4 months of treatment (T2) correspond to PC-caloric avoidance factor scores at T1 and T2, respectively. Successful high-fat food reintroduction was defined as the presence of avoidance at T1 and the absence of avoidance at T2 for at least one high-fat food (butter, fries, cheese, pastries, or cold meats). Factors were assessed at T1. The left set of arrows therefore depicts associations between high-calorie food avoidance and factors at T1, while the right set of arrows indicates factors at T1 which predict high-calorie food avoidance or successful high-fat food reintroduction at T2. Asterisks indicate factors which covary with high-calorie food avoidance between T1 and T2. Arrow thickness reflects *p*-values obtained in the multiple regressions. *AN* anorexia nervosa, *BMI* body mass index, *EAT* Eating Attitudes Test Total Score, *HADS* Hospital Anxiety and Depressive Scale, *p* *p*-value, *PANAS* positive and negative affect schedule, *WSAS* Work and Social Adjustment Scale

PC-caloric avoidance factor score at T1 positively correlated with minimum lifetime BMI (*r* = 0.308, *p* < 0.001), perceived BMI (*r* = 0.226, *p* = 0.010) and perceptual distortion (*r* = 0.232, *p* = 0.008), and with EAT total (*r* = 0.520, *p* < 0.001), HADS anxiety (*r* = 0.230, *p* = 0.008) and PANAS negative (*r* = 0.211, *p* = 0.016) scores. It negatively correlated with the difference between current and minimum lifetime BMI (*r* = − 0.286, *p* = 0.002; Table 1). A linear regression indicated a significant effect of EAT total score (Wald χ^2^ = 20.412, *p* < 0.001) and minimum lifetime BMI (Wald χ^2^ = 4.070, *p* = 0.046; Fig. 1).

**Table 1:** Factors correlated to, or associated with, factor scores of food avoidance in 130 patients with anorexia nervosa at baseline

Patients’ characteristics	PC-caloric avoidance factor score at T2			PC-animal avoidance factor score at T2			PC-vegetal avoidance factor score at T2		
	*r*	*U*	*p*	*r*	*U*	*p*	*r*	*U*	*p*
Age	− 0.106		0.229	− 0.124		0.159	0.089		0.316
Education		1789	0.308		1633	0.077		1861	0.500
Working		1003	0.837		1016	0.911		961	0.610
Familial history of ED		1432	0.187		1536	0.440		1680	0.987
Subtype		2010	0.772		2064	0.972		1516	0.009
Age at onset	− 0.105		0.234	− 0.173		0.049	− 0.008		0.927
Illness duration	− 0.068		0.444	− 0.053		0.546	0.105		0.233
Current BMI	0.008		0.925	− 0.007		0.939	0.092		0.295
Minimum lifetime BMI	0.308		**< 0.001**	0.251		**0.007**	0.195		0.037
Maximum lifetime BMI	0.120		0.201	0.022		0.818	0.077		0.410
BMI max-current	0.104		0.270	0.002		0.983	0.042		0.652
BMI current-min	− 0.286		**0.002**	− 0.231		**0.013**	− 0.131		0.162
BMI max–min	− 0.037		0.696	− 0.108		0.252	− 0.022		0.816
Perceived BMI	0.226		**0.010**	0.263		**0.003**	0.028		0.753
Perceptual distortion	0.232		**0.008**	0.275		**0.002**	− 0.047		0.594
Subjective ideal BMI	− 0.092		0.321	− 0.253		**0.006**	− 0.053		0.565
EAT Total	0.520		**< 0.001**	0.408		**< 0.001**	0.263		0.003
EAT Dieting	0.557		**< 0.001**	0.441		**< 0.001**	0.230		0.008
EAT Bulimia	0.266		**0.002**	0.191		0.029	0.190		0.031
EAT Oral	0.325		**< 0.001**	0.260		**0.003**	0.221		0.011
HADS anxiety score	0.230		**0.008**	0.285		**0.001**	0.213		0.015
HADS anxiety syndrome		653	0.128		692	0.215		827	0.799
HADS depression score	0.100		0.258	0.172		0.051	0.089		0.314
HADS depression syndrome		1536	0.037		1579	0.061		1776	0.356
PANAS positive	0.005		0.953	− 0.128		0.148	− 0.030		0.734
PANAS negative	0.211		**0.016**	0.245		**0.005**	0.197		0.025
WSAS	0.165		0.061	0.081		0.359	0.137		0.120

*BMI* body mass index, *EAT* eating attitudes test-26, *ED* eating disorder, *HADS* Hospital Anxiety and Depressive Scale, *p* *p*-value, *PANAS* positive and negative affect schedule, *PC* principal component, *r* Pearson’s *r*, *U* Mann–Whitney *U*, *WSAS* Work and Social Adjustment Scale. Bold numbers indicate significant *p*-values after implementation of the Benjamini–Hochberg procedure

PC-animal avoidance factor score at T1 negatively correlated with subjective ideal BMI (*r* = − 0.253, *p* = 0.006). It positively correlated with minimum lifetime BMI (*r* = 0.251, *p* = 0.007), perceived BMI (*r* = 0.263, *p* = 0.003), perceptual distortion (*r* = 0.275, *p* = 0.002), EAT total (*r* = 0.408, *p* < 0.001), HADS anxiety (*r* = 0.285, *p* = 0.001) and PANAS negative (*r* = 0.245, *p* = 0.005) scores (Table 1). Effects of minimum lifetime BMI (Wald χ^2^ = 11.526, *p* < 0.001) and perceptual distortion (Wald χ^2^ = 7.037, *p* = 0.009) remained significant in the linear regression, while a tendency was observed for subjective ideal BMI (Wald χ^2^ = 3.553, *p* = 0.062).

None of the correlations with PC-vegetal avoidance factor score at T1 was significant (Table 1).

### Evolution between T1 and T2

Factor scores, current BMI, perceived BMI, and EAT, HADS, PANAS, and WSAS scores all significantly evolved between T1 and T2 (Table S2).

### Predicting factors of food avoidance

We identified predicting factors of food avoidance through Pearson’s correlations between factor scores at T2 and patients’ clinical characteristics at T1 (Table 2).

**Table 2:** Predicting factors of food avoidance

Patients’ characteristics at T1	PC-caloric avoidance factor score at T2			PC-animal avoidance factor score at T2			PC-vegetal avoidance factor score at T2		
	*r*	*U*	*p*	*r*	*U*	*p*	*r*	*U*	*p*
Age	− 0.088		0.321	− 0.097		0.273	0.049		0.581
Education		1957	0.829		1924	0.708		1659	0.100
Working		1030	0.991		1011	0.882		911	0.383
Familial history of ED		1622	0.748		1527	0.412		1387	0.120
Subtype		1773	0.160		1740	0.119		1648	0.046
Age at onset	− 0.263		**0.003**	− 0.308		**< 0.001**	− 0.070		0.426
Illness duration	0.034		0.700	0.047		0.598	0.092		0.299
Current BMI	0.014		0.879	− 0.008		0.932	0.082		0.352
Minimum lifetime BMI	0.144		0.126	0.124		0.186	0.162		0.083
Maximum lifetime BMI	0.159		0.089	− 0.050		0.596	− 0.014		0.879
BMI max–current	0.151		0.107	− 0.070		0.456	− 0.052		0.579
BMI current–min	− 0.118		0.209	− 0.101		0.281	− 0.103		0.274
BMI max–min	0.088		0.351	− 0.115		0.222	− 0.098		0.295
Perceived BMI	0.257		**0.003**	0.209		**0.017**	− 0.009		0.915
Perceptual distortion	0.241		**0.006**	0.220		**0.012**	− 0.065		0.462
Subjective ideal BMI	− 0.193		0.036	− 0.243		**0.008**	− 0.005		0.955
EAT Total	0.434		**< 0.001**	0.263		**0.002**	0.091		0.303
EAT Dieting	0.465		**< 0.001**	0.282		**0.001**	0.084		0.342
EAT Bulimia	0.242		**0.006**	0.172		0.050	0.096		0.278
EAT Oral	0.250		**0.004**	0.130		0.142	0.040		0.652
HADS anxiety score	0.281		**0.001**	0.211		**0.016**	− 0.013		0.884
HADS anxiety syndrome		622	0.080		856	0.965		827	0.799
HADS depression score	0.098		0.266	0.141		0.109	0.119		0.179
HADS depression syndrome		1605	0.080		1693	0.185		1594	0.072
PANAS positive	− 0.075		0.394	− 0.217		**0.013**	− 0.146		0.098
PANAS negative	0.276		**0.001**	0.140		0.112	− 0.119		0.178
WSAS	0.212		**0.016**	0.095		0.282	0.042		0.635

*BMI* body mass index, *EAT* eating attitudes test-26, *ED* eating disorder, *HADS* Hospital Anxiety and Depressive Scale, *p* *p*-value, *PANAS* positive and negative affect schedule, *PC* principal component, *r* Pearson’s *r*, *T1* at baseline, *T2* after 4 months of treatment, *U* Mann–Whitney *U*, *WSAS* Work and Social Adjustment Scale. Bold numbers indicate significant *p*-values after implementation of the Benjamini–Hochberg procedure

Avoidance of high-calorie foods at T2 was predicted by higher EAT (*r* = 0.434, *p* < 0.001), HADS anxiety (*r* = 0.281, *p* = 0.001), PANAS negative (*r* = 0.276, *p* = 0.001) and WSAS (*r* = 0.212, *p* = 0.016) scores at T1. It was also predicted by a younger age at onset (*r* = − 0.263, *p* = 0.003), a higher perceived BMI (*r* = 0.257, *p* = 0.003) and more perceptual distortion (*r* = 0.241, *p* = 0.006; Table 2). Results from the linear regression indicated a significant effect of EAT total score (Wald χ^2^ = 14.458, *p* < 0.001) and age at onset (Wald χ^2^ = 8.662, *p* = 0.004; Fig. 1).

Avoidance of animal-based foods at T2 was predicted by higher EAT (*r* = 0.263, *p* = 0.002), and HADS anxiety (*r* = 0.211, *p* = 0.016) scores, and by a lower PANAS positive score (*r* = − 0.217, *p* = 0.013). It was also predicted by a younger age at onset (*r* = − 0.308, *p* < 0.001), a lower subjective ideal BMI (*r* = − 0.243, *p* = 0.008), a higher perceived BMI (*r* = 0.209, *p* = 0.017) and more perceptual distortion (*r* = 0.220, *p* = 0.012; Table 2). Age at onset (Wald χ^2^ = 4.642, *p* = 0.033) and EAT total score (Wald χ^2^ = 4.213, *p* = 0.042) retained a significant effect in the linear regression.

No factor significantly predicted avoidance of fruits and vegetables at T2 (Table 2).

### Improvement of avoidance

Factor scores covaried with different variables. Greater reduction of PC-caloric avoidance factor score was strongly associated with greater BMI increase (*r* = − 0.227, *p* = 0.009) and greater reductions of perceptual distortion (*r* = 0.252, *p* = 0.004) and EAT score (*r* = 0.383, *p* < 0.001). A greater reduction of PC-caloric avoidance factor score was observed in patients in remission of depression (*U* = 1150, *p* = 0.011). Only the effect of EAT score remained significant in the linear regression (Wald χ^2^ = 10.980, *p* = 0.001). Greater reduction of PC-animal avoidance factor score strongly correlated with a greater reduction of EAT score (*r* = 0.324, *p* < 0.001). PC-vegetal avoidance factor score did not covary with any factors (Fig. 1; Table 3).

**Table 3:** Clinical characteristics covarying with factor scores of food avoidance between two visits of 130 patients with anorexia nervosa

Patients’ characteristics (T2–T1)	PC-caloric avoidance factor score difference (T2–T1)			PC-animal avoidance factor score difference (T2–T1)			PC-vegetal avoidance factor score difference (T2–T1)		
	*r*	*U*	*p*	*r*	*U*	*p*	*r*	*U*	*p*
Age	0.028		0.754	0.031		0.727	− 0.034		0.700
Current BMI	− 0.227		**0.009**	− 0.071		0.419	0.172		0.050
Perceived BMI	0.075		0.396	0.019		0.827	0.035		0.691
Perceptual distortion	0.252		**0.004**	0.065		0.466	− 0.100		0.257
EAT Total	0.383		**< 0.001**	0.324		**< 0.001**	0.125		0.157
EAT Dieting	0.418		**< 0.001**	0.351		**< 0.001**	0.088		0.320
EAT Bulimia	0.255		**0.003**	0.230		**0.008**	0.119		0.177
EAT Oral	0.198		0.024	0.161		0.068	0.118		0.181
HADS anxiety score	0.161		0.067	0.155		0.079	0.043		0.629
Anxiety remission		826	0.078		910	0.221		1084	0.920
HADS depression score	0.182		0.039	0.103		0.245	− 0.044		0.618
Depression remission		1150	**0.011**		1334	0.115		1588	0.818
PANAS positive	− 0.042		0.633	− 0.073		0.410	− 0.078		0.381
PANAS negative	0.131		0.137	0.200		0.022	0.146		0.098
WSAS	0.071		0.424	0.119		0.176	0.099		0.262
Delay between visits	0.024		0.784	0.072		0.417	− 0.059		0.509

*BMI* body mass index, *EAT* eating attitudes test-26, *HADS* Hospital Anxiety and Depressive Scale, *p* *p*-value, *PANAS* positive and negative affect schedule, *PC* principal component, *r* Pearson’s *r*, *T1* at baseline, *T2* after 4 months of treatment, *U* Mann–Whitney *U*, *WSAS* Work and Social Adjustment Scale. Bold numbers indicate significant *p*-values after implementation of the Benjamini–Hochberg procedure

Patients who successfully reintroduced at least one high-fat food had, at T1, lower HADS anxiety (*U* = 1243, *p* = 0.007) and EAT bulimia (*U* = 1222, *p* = 0.005) scores. EAT oral score also improved more in patients who successfully reintroduced at least one high-fat food than in those who did not (*U* = 1215, *p* = 0.004; Table 4). EAT oral score (Wald χ^2^ = 7.941, *p* = 0.005) and HADS anxiety score (Wald χ^2^ = 3.883, *p* = 0.049) retained a significant effect in the logistic regression (Fig. 1).

**Table 4:** Characteristics of 130 patients with anorexia nervosa who reintroduced at least one high-fat food (versus did not) after 4 months of treatment

	Successful food reintroduction (*N* = 39)			No food reintroduction (*N* = 91)			Statistics		
	Mean	SD	%	Mean	SD	%	*Χ*^2^	*U*	*p*
Age	24.18	9.80		25.47	11.37			1604	0.386
Education (high)			30.8			44.4	2.11		0.146
Working (presently)			22.6			21.5	0.01		0.903
Familial history of ED (yes)			23.1			31.5	0.93		0.336
Subtype (restrictive)			69.2			52.8	3.00		0.083
Age at onset	18.21	6.72		16.78	3.78			1726	0.807
Illness duration	5.97	6.54		8.69	10.50			1510	0.178
BMI									
At T1	15.48	1.93		15.23	1.88			1609	0.402
Difference	1.15	1.67		1.07	1.68			1707	0.734
Minimum lifetime BMI	13.28	1.58		13.40	1.82			1325	0.559
Maximum lifetime BMI	21.11	3.62		21.29	3.36			1345	0.642
BMI max-current	5.56	3.41		5.95	3.12			1274	0.372
BMI current-min	2.28	1.79		1.94	1.50			1322	0.548
BMI max–min	7.84	3.62		7.89	3.27			1308	0.494
Perceived BMI									
At T1	19.46	2.82		19.71	3.20			1734	0.838
Difference	1.28	2.30		0.79	2.51			1571	0.298
Perceptual distortion									
At T1	1.27	0.20		1.30	0.21			1556	0.268
Difference	− 0.01	0.15		− 0.03	0.17			1474	0.127
Subjective ideal BMI	17.69	1.77		17.28	2.06			1341	0.377
EAT total									
At T1	30.54	17.54		37.00	15.73			1383	0.047
Difference	− 12.90	12.58		− 6.54	12.85			1288	0.013
EAT dieting									
At T1	16.00	10.85		19.75	10.05			1413	0.066
Difference	− 6.54	7.94		− 3.41	7.35			1370	0.040
EAT bulimia									
At T1	6.23	4.39		8.51	4.42			1222	**0.005**
Difference	− 2.46	3.75		− 1.36	3.49			1497	0.155
EAT oral									
At T1	8.31	4.70		8.75	5.00			1693	0.678
Difference	− 3.90	3.62		− 1.77	4.71			1215	**0.004**
HADS anxiety									
At T1	11.74	4.25		13.96	3.92			1243	**0.007**
Remission			25.6			11.0	4.50		0.034
HADS depression									
At T1	8.49	4.13		9.29	3.55			1548	0.249
Remission			28.2			25.3	0.12		0.728
PANAS positive									
At T1	29.23	6.84		29.26	6.87			1709	0.741
Difference	2.87	5.93		0.71	6.08			1489	0.147
PANAS negative									
At T1	33.64	8.73		37.00	7.34			1360	0.035
Difference	− 4.79	7.42		− 3.03	8.15			1542	0.238
WSAS									
At T1	21.56	8.26		24.23	8.06			1408	0.062
Difference	− 4.85	9.31		− 4.01	10.33			1711	0.747
Delay between visits	126.13	89.08		136.21	101.77			1719	0.778

*BMI* body mass index, *EAT* eating attitudes test-26, *ED* eating disorder, *HADS* Hospital Anxiety and Depressive Scale, *p* *p*-value, *PANAS* positive and negative affect schedule, *SD* standard deviation, *T1* at baseline, *U* Mann–Whitney *U*, *WSAS* Work and Social Adjustment Scale, *χ*^*2*^ Chi-squared test. Bold numbers indicate significant *p*-values after implementation of the Benjamini–Hochberg procedure

### Cross-lagged panel models

**Fig. 2:**
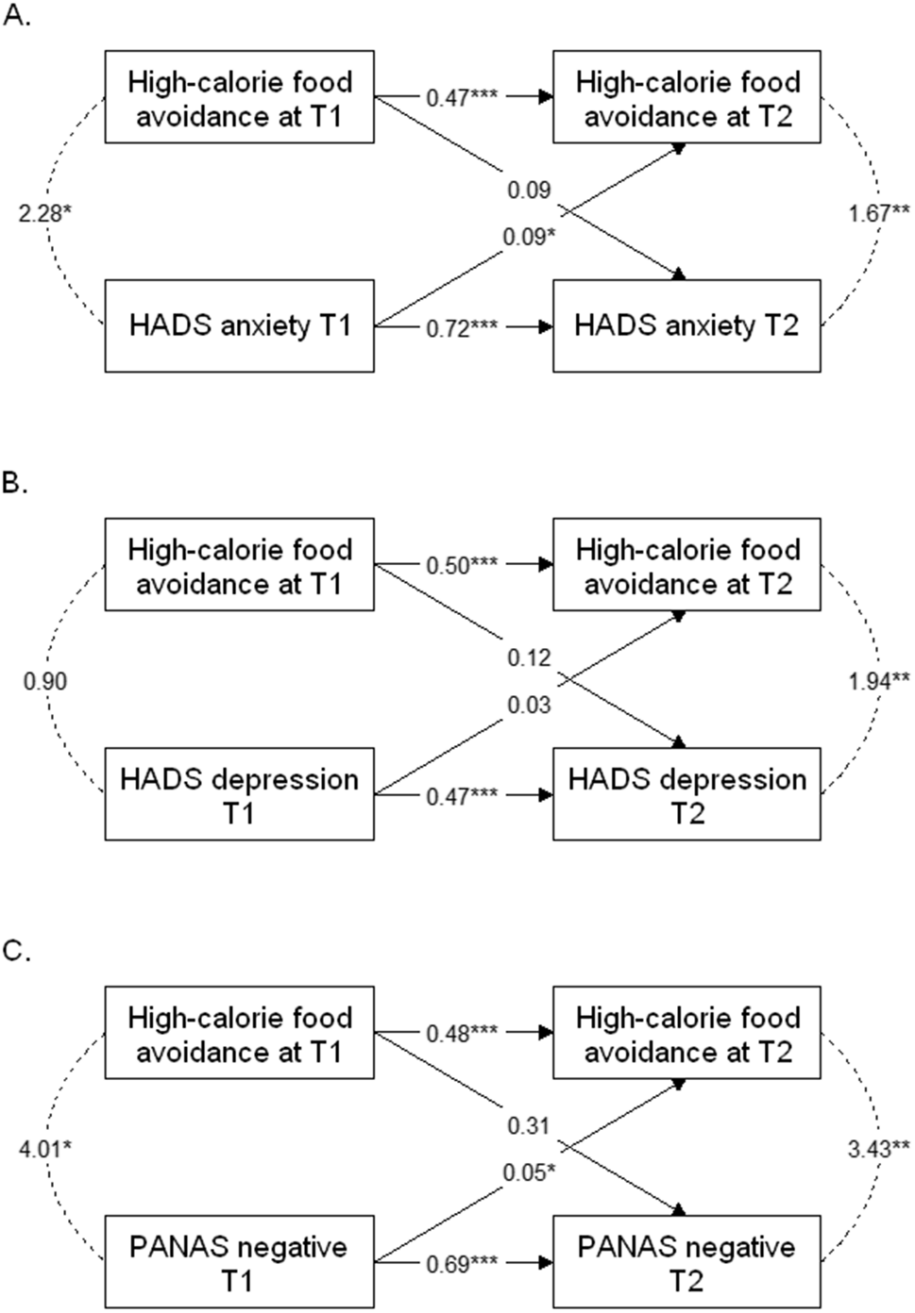
Cross-lagged panel models depicting the causal relationship between high-calorie food avoidance and **A** anxiety, **B** depression, and **C** negative affect. These models depict the synchronous correlations between the two variables at T1 and at T2, the autoregressive paths of each variable between T1 and T2, and the cross-lagged paths. Numbers indicate regression coefficients. ***p < 0.001, **p < 0.01, *p < 0.05

Because avoidance factor scores correlate with other variables at both T1 and T2, we performed exploratory analyses to further investigate the causal relationship between high-calorie food avoidance and variables of interest, namely anxiety, depression and negative affect (Fig. 2). The cross-lagged paths suggest that anxiety and negative affect cause food avoidance, and not the opposite. For depression, the relationship was not significant, suggesting that food avoidance and depression do not cause each other.

## Discussion

After distributing food avoidance in three domains (high calorie, animal-based and vegetal), we found that food avoidance, especially for high-calorie foods, was associated with the clinical severity of AN (as indicated by EAT scores) and with mood and anxiety dimensions. A younger age at onset was associated with the maintenance of food avoidance. We also observed associations between food avoidance and BMI- and body image-related factors such as minimum lifetime BMI, subjective ideal BMI and perceptual distortion.

Correlations between food avoidance and EAT scores confirm that food avoidance is associated with AN severity and validate our ad hoc questionnaire. These correlations were expected as food restriction typically results from the concerns about weight which are characteristic of AN [28]. Food restriction can be quantitative or qualitative: while patients can exclude certain foods from their diet (qualitative restriction), they can also restrict the amount of food they eat without reducing diet diversity (quantitative restriction) [29]. Qualitative restriction is the focus of the present study, as we assess the avoidance of specific food items. We observed that, in patients who reintroduced at least one high-fat food into their diet, EAT scores were lower at baseline and improved more during treatment, once again confirming that food avoidance is related to illness severity.

We found that a younger age at onset was associated with more maintenance of food avoidance. This observation concurs with the literature, as a younger age at onset is known to be associated with more severe symptomatology [30, 31]. The impact of age at onset on illness severity may stem from the aetiology of AN, which involves a complex interplay between genetics and the environment [32] and differs depending on age at onset; indeed, early- and typical-onset AN show distinct genetic correlation patterns with risk factors for the disease [33].

While the multiple regressions did not highlight these results, we initially observed an association between food avoidance and anxiety, depression and negative affect. Anxiety and mood disorders are common comorbidities in AN [34]. The causal link between food avoidance and mood disorder has not been clearly established: depression may promote food avoidance while food avoidance, e.g. depriving oneself of high-fat foods, may worsen depressive symptoms. Likewise, it is not clear whether anxiety triggers food avoidance or the opposite. On one hand, fastidiously screening the caloric content of foods and avoiding the consumption of calorie-rich foods is a strategy of patients with AN to inefficiently alleviate their anxiety [3]. On the other hand, food avoidance may contribute to anxiety and depression: because of reduced dietary intake, micronutrient status is often altered in patients with AN [11, 35]. Some of the most frequent deficiencies are vitamin B9 and selenium deficits. Both of these elements are essential for neuronal function, and their deficiencies have been linked to depression and anxiety [11, 36]. Such correlations between AN severity, nutritional status, and anxiety and depression, are at the origin of the conceptualization of AN as a metabo-psychiatric disorder [37]. Our additional exploratory analyses using cross-lagged panel models suggested that anxiety and negative affect caused high-calorie food avoidance, and not the opposite. Depression correlated with high-calorie food avoidance at T2 but the cross-lagged paths were not significant, suggesting that depression is not an actor in the maintenance of food avoidance. This implies that, during treatment, focusing on reducing anxiety and negative affect (but not depression) may be a way to indirectly reduce food avoidance.

Our results also indicated that food avoidance was associated with BMI- and body image-related factors. Correlations with perceived BMI and perceptual distortion make sense since body image distortion is one of the core characteristics of AN [2]. The association of lower minimum lifetime BMI with lower levels of food avoidance was more surprising, as a lower minimum lifetime BMI indicates more severe AN. In an attempt to explain our finding, we examined the difference between current and minimum lifetime BMI, because patients were enrolled in our study at different stages of illness and the difference between current and minimum lifetime BMI can reflect the benefit of care, i.e. weight gain. We observed that a bigger difference between current and minimum lifetime BMI was indeed associated with less food avoidance. Taken together, this suggests that food avoidance could depend not only on illness severity but also on recovery status. In other words, levels of food avoidance were not lower in patients whose minimum BMI were less severe, but in those who regained some weight. To confirm this theory, it would have been interesting to have more accurate information about the time trend of patients’ illness and weight history.

### Strengths and limits

We hereby present a longitudinal and multicentric study conducted in a sample including both teenagers and adults. This study focuses on a topic which, although central in AN, is understudied, namely food avoidance and more specifically its qualitative dimension. It is understudied to such an extent that no consensual questionnaire assesses food avoidance, hence the need to build one. Our ad hoc questionnaire allowed to detect expected time changes and correlated with illness severity, suggesting that it is a pertinent tool. A potential limitation of this tool is its subjectivity, especially as insight is impaired in AN [13]. To mitigate this potential bias, it could be interesting to combine this questionnaire to physiological measures such as skin conductance response or pupil size. Another aspect to consider is that our sample was characterized by a wide range of age and illness duration, as it included both adolescents, young adults and older adults (up to 52 years old), while length of the disorder ranged between a couple of months and 34 years. While this variability is a strength (better representativeness), it can also be a limitation. However, in our analyses, we did not find any significant effects of age or illness duration. We performed additional analyses (Table S3) in which we ran our main analyses again, this time in adolescents and adults separately. Associated factors were similar in both groups. Overall, fewer factors were significant in each group than in the whole sample, but this may be due to the reduced statistical power caused by the smaller sample sizes. Other limitations can be considered. Firstly, principal components are not always easy to interpret. Factor scores were computed from the 16 ratings of food avoidance and therefore relied on indirect measures. Nevertheless, component loadings clearly indicated that our three principal components reflected avoidance of high-calorie foods, animal-based foods, and fruits and vegetables, respectively, which corresponds to a relatively simple and intuitive classification. Secondly, the variables included in our regression analyses were not independent. This multicollinearity, although limited (variance inflation factor VIF < 2), may weaken the statistical power of our regression models. Thirdly, a control group could have helped identify factors and associations that are characteristic of AN. Fourthly, some additional measures could have brought some interesting information. Food restriction can be qualitative or quantitative, but our measures only assess self-reported qualitative food restriction, and we do not have data about the nutritional status of patients and the reality of food avoidance. Also, our body image perception test uses drawn silhouettes that are rather minimalistic and may not be as ecologically valid as tests using patients’ own silhouettes like in other studies [38], although the present test is less constraining. Finally, it is interesting to note that, as food choices are impacted by the sociocultural context (e.g. cultural values, lifestyles, food movements) [39], our sample exclusively made of French female outpatients may not be representative of all patients with AN.

### What is already known on this subject?

Food avoidance in AN can consist not only in reducing the amount of ingested food, but also in limiting diet diversity. This disordered eating behaviour is responsible for unmet nutrient needs and is a challenge in the treatment of AN, but research on the topic is scarce.

### What this study adds?

- This prospective longitudinal and multicentre study assesses food avoidance in teenagers and adults with AN before and after 4 months of care.
- Even though qualitative food avoidance is not a diagnostic criterion for AN, the present study shows that it could be an informative indicator of AN severity.
- It also suggests that improving anxiety or negative affect may be a leverage to reduce food avoidance.

## Supplementary Information

Below is the link to the electronic supplementary material.

Supplementary file1 (DOCX 110 KB)

## Data Availability

The data generated and analysed during the current study are available from the corresponding author on request.
