# Supplementary material for: Food avoidance in anorexia nervosa: associated and predicting factors
Source: Eat Weight Disord. 2023 Feb 23;28(1):24. doi: 10.1007/s40519-023-01545-4 (PMC9950187; doi:10.1007/s40519-023-01545-4)
Supplement: Supplementary file 1 — Supplementary file1 (DOCX 110 KB) [file 40519_2023_1545_MOESM1_ESM.docx]

**Supplementary information**

| **Table S1** |  |  |  |
| --- | --- | --- | --- |
| Component loadings of food avoidance at T1 on principal components | | | |
|  | PC-caloric | PC-animal | PC-produce |
| Butter | 0.74248 | 0.07192 | -0.18319 |
| Starchy foods | 0.53053 | 0.39361 | 0.30646 |
| Fries | 0.65886 | 0.07053 | -0.17651 |
| Cheese | 0.50484 | 0.15960 | 0.02711 |
| Pastries | 0.74884 | 0.04063 | -0.01044 |
| Cold meats | 0.62821 | 0.16305 | -0.23682 |
| Ham | 0.23015 | 0.69641 | 0.01444 |
| Red meat | 0.25967 | 0.75917 | -0.00838 |
| White meat | 0.05148 | 0.86431 | -0.02049 |
| White fish | -0.00888 | 0.58036 | 0.36411 |
| 0% fat dairy produce | -0.20401 | -0.01948 | 0.28878 |
| Green vegetables | -0.03246 | -0.01435 | 0.80784 |
| Tomatoes | 0.04997 | -0.00141 | 0.76142 |
| Fresh fruits | -0.06079 | 0.20134 | 0.61626 |
| Dried fruits | 0.57902 | 0.07471 | 0.22970 |
| Whole wheat bread | 0.48238 | 0.19317 | 0.46406 |
| *Cells are greyed out when component loadings are higher than 0.5* | | | |

| **Table S2** |  |  |  |  |  |  |  |  |  |  |  |
| --- | --- | --- | --- | --- | --- | --- | --- | --- | --- | --- | --- |
| Clinical characteristics of 130 patients with anorexia nervosa seen four months apart. | | | | | | | | | | | |
| Clinical characteristics | At baseline | | |  | After 4 months of treatment | | |  | Statistics | | |
|  | Mean | SD | % |  | Mean | SD | % |  | ꭓ2 | Wilcoxon | p |
| PC-caloric factor score | 5.96 | 2.42 |  |  | 5.49 | 2.23 |  |  |  | 5750 | **<0.001** |
| PC-animal factor score | 3.21 | 1.88 |  |  | 2.75 | 1.91 |  |  |  | 5730 | **<0.001** |
| PC-produce factor score | 0.57 | 1.24 |  |  | 0.40 | 1.29 |  |  |  | 5510 | **0.004** |
| Actual BMI | 15.31 | 1.89 |  |  | 16.40 | 2.31 |  |  |  | 954 | **<0.001** |
| Perceived BMI | 19.64 | 3.09 |  |  | 20.58 | 3.12 |  |  |  | 1257 | **<0.001** |
| Perceptual distortion | 1.29 | 0.21 |  |  | 1.27 | 0.21 |  |  |  | 4543 | 0.136 |
| EAT Total | 35.06 | 16.50 |  |  | 26.62 | 17.31 |  |  |  | 6382 | **<0.001** |
| EAT Dieting | 18.62 | 10.39 |  |  | 14.28 | 10.27 |  |  |  | 5650 | **<0.001** |
| EAT Bulimia | 7.82 | 4.51 |  |  | 6.13 | 4.97 |  |  |  | 3950 | **<0.001** |
| EAT Oral | 8.62 | 4.90 |  |  | 6.21 | 4.74 |  |  |  | 5385 | **<0.001** |
| HADS anxiety score | 13.29 | 4.13 |  |  | 11.67 | 4.67 |  |  |  | 5093 | **<0.001** |
| HADS anxiety syndrome |  |  | 88.5% |  |  |  | 78.5% |  | 10.1 |  | **0.001** |
| HADS depression score | 9.05 | 3.74 |  |  | 7.48 | 4.02 |  |  |  | 5249 | **<0.001** |
| HADS depression syndrome |  |  | 63.1% |  |  |  | 46.9% |  | 12.0 |  | **<0.001** |
| PANAS positive | 29.25 | 6.84 |  |  | 30.62 | 6.95 |  |  |  | 2779 | **0.013** |
| PANAS negative | 35.99 | 7.90 |  |  | 32.43 | 9.43 |  |  |  | 5843 | **<0.001** |
| WSAS | 23.43 | 8.21 |  |  | 19.32 | 10.45 |  |  |  | 5368 | **<0.001** |
| *BMI: body mass index; EAT: eating attitudes test-26; HADS: hospital anxiety and depressive scale; p: p-value; PANAS: positive and negative affect schedule; PC: principal component; SD: standard deviation; WSAS: work and social adjustment scale; ꭓ2: chi-squared test. Bold numbers indicate significant p-values (p<0.05).* | | | | | | | | | | | |

**Table S3. Analyses performed separately in adolescents (age ≤ 20) and in adults (age > 20)**

| **Table S3.1. Clinical characteristics of patients with anorexia nervosa at baseline and after 4 months of treatment, described for the whole sample (N=130), and separately for adolescents (age≤20, N=54) and adults (age>20, N=76).** | | | | | | | | | | | |
| --- | --- | --- | --- | --- | --- | --- | --- | --- | --- | --- | --- |
| Clinical characteristics | At baseline | | |  | After 4 months of treatment | | |  | Statistics | | |
|  | Mean | SD | % |  | Mean | SD | % |  | ꭓ2 | Wilcoxon | p |
| PC-caloric factor score | 5.96 | 2.42 |  |  | 5.49 | 2.23 |  |  |  | 5750 | **<0.001** |
| Adolescents | 6.19 | 2.41 |  |  | 5.62 | 2.36 |  |  |  | 1050 | **0.008** |
| Adults | 5.79 | 2.43 |  |  | 5.39 | 2.15 |  |  |  | 1926 | **0.017** |
| PC-animal factor score | 3.21 | 1.88 |  |  | 2.75 | 1.91 |  |  |  | 5730 | **<0.001** |
| Adolescents | 3.39 | 1.84 |  |  | 2.90 | 2.03 |  |  |  | 1072 | **0.005** |
| Adults | 3.08 | 1.91 |  |  | 2.65 | 1.82 |  |  |  | 1866 | **0.037** |
| PC-produce factor score | 0.57 | 1.24 |  |  | 0.40 | 1.29 |  |  |  | 5510 | **0.004** |
| Adolescents | 0.49 | 1.24 |  |  | 0.47 | 1.40 |  |  |  | 835 | 0.428 |
| Adults | 0.62 | 1.25 |  |  | 0.35 | 1.22 |  |  |  | 2063 | **0.002** |
| Actual BMI | 15.31 | 1.89 |  |  | 16.40 | 2.31 |  |  |  | 954 | **<0.001** |
| Adolescents | 15.06 | 1.78 |  |  | 16.24 | 2.27 |  |  |  | 125 | **<0.001** |
| Adults | 15.48 | 1.96 |  |  | 16.52 | 2.35 |  |  |  | 417 | **<0.001** |
| Perceived BMI | 19.64 | 3.09 |  |  | 20.58 | 3.12 |  |  |  | 1257 | **<0.001** |
| Adolescents | 19.73 | 3.18 |  |  | 20.97 | 3.14 |  |  |  | 172 | **<0.001** |
| Adults | 19.57 | 3.04 |  |  | 20.30 | 3.09 |  |  |  | 498 | **0.014** |
| Perceptual distortion | 1.29 | 0.21 |  |  | 1.27 | 0.21 |  |  |  | 4543 | 0.136 |
| Adolescents | 1.31 | 0.20 |  |  | 1.30 | 0.20 |  |  |  | 795 | 0.339 |
| Adults | 1.27 | 0.21 |  |  | 1.24 | 0.20 |  |  |  | 1562 | 0.246 |
| EAT Total | 35.06 | 16.50 |  |  | 26.62 | 17.31 |  |  |  | 6382 | **<0.001** |
| Adolescents | 35.30 | 17.39 |  |  | 27.43 | 18.14 |  |  |  | 1038 | **<0.001** |
| Adults | 34.90 | 15.95 |  |  | 26.04 | 16.80 |  |  |  | 2291 | **<0.001** |
| EAT Dieting | 18.62 | 10.39 |  |  | 14.28 | 10.27 |  |  |  | 5650 | **<0.001** |
| Adolescents | 20.26 | 10.93 |  |  | 15.76 | 10.99 |  |  |  | 1008 | **<0.001** |
| Adults | 17.46 | 9.91 |  |  | 13.22 | 9.66 |  |  |  | 1902 | **<0.001** |
| EAT Bulimia | 7.82 | 4.51 |  |  | 6.13 | 4.97 |  |  |  | 3950 | **<0.001** |
| Adolescents | 7.06 | 3.85 |  |  | 5.98 | 4.43 |  |  |  | 576 | 0.060 |
| Adults | 8.37 | 4.88 |  |  | 6.24 | 5.34 |  |  |  | 1525 | **<0.001** |
| EAT Oral | 8.62 | 4.90 |  |  | 6.21 | 4.74 |  |  |  | 5385 | **<0.001** |
| Adolescents | 7.98 | 5.05 |  |  | 5.69 | 4.72 |  |  |  | 891 | **<0.001** |
| Adults | 9.07 | 4.77 |  |  | 6.58 | 4.76 |  |  |  | 1925 | **<0.001** |
| HADS anxiety score | 13.29 | 4.13 |  |  | 11.67 | 4.67 |  |  |  | 5093 | **<0.001** |
| Adolescents | 13.46 | 4.47 |  |  | 12.02 | 5.04 |  |  |  | 898 | **0.004** |
| Adults | 13.17 | 3.90 |  |  | 11.42 | 4.40 |  |  |  | 1736 | **<0.001** |
| HADS anxiety syndrome |  |  | 88.5% |  |  |  | 78.5% |  | 10.1 |  | **0.001** |
| Adolescents |  |  | 85.2% |  |  |  | 75.9% |  | 7.59 |  | **0.006** |
| Adults |  |  | 90.8% |  |  |  | 80.3% |  | 2.6 |  | 0.107 |
| HADS depression score | 9.05 | 3.74 |  |  | 7.48 | 4.02 |  |  |  | 5249 | **<0.001** |
| Adolescents | 9.04 | 3.94 |  |  | 7.63 | 4.19 |  |  |  | 841 | **0.023** |
| Adults | 9.05 | 3.61 |  |  | 7.37 | 3.92 |  |  |  | 1912 | **<0.001** |
| HADS depression syndrome |  |  | 63.1% |  |  |  | 46.9% |  | 12 |  | **<0.001** |
| Adolescents |  |  | 68.5% |  |  |  | 50.0% |  | 10.4 |  | **0.001** |
| Adults |  |  | 59.2% |  |  |  | 44.7% |  | 3.3 |  | 0.069 |
| PANAS positive | 29.25 | 6.84 |  |  | 30.62 | 6.95 |  |  |  | 2779 | **0.013** |
| Adolescents | 27.50 | 6.54 |  |  | 29.93 | 6.67 |  |  |  | 339 | **0.004** |
| Adults | 30.50 | 6.81 |  |  | 31.11 | 7.14 |  |  |  | 1186 | 0.472 |
| PANAS negative | 35.99 | 7.90 |  |  | 32.43 | 9.43 |  |  |  | 5843 | **<0.001** |
| Adolescents | 35.89 | 8.50 |  |  | 32.17 | 10.28 |  |  |  | 1058 | **<0.001** |
| Adults | 36.07 | 7.50 |  |  | 32.62 | 8.83 |  |  |  | 1953 | **< 0.001** |
| WSAS | 23.43 | 8.21 |  |  | 19.32 | 10.45 |  |  |  | 5368 | **<0.001** |
| Adolescents | 22.94 | 8.57 |  |  | 17.76 | 10.35 |  |  |  | 1058 | **< 0.001** |
| Adults | 23.79 | 7.98 |  |  | 20.44 | 10.44 |  |  |  | 1701 | **0.016** |
| *BMI: body mass index; EAT: eating attitudes test-26; HADS: hospital anxiety and depressive scale; p: p-value; PANAS: positive and negative affect schedule; PC: principal component; SD: standard deviation; WSAS: work and social adjustment scale; ꭓ2: chi-squared test. Bold numbers indicate significant p-values (p<0.05).* | | | | | | | | | | | |

| **Table S3.2. Factors correlated to, or associated with, factor scores of food avoidance in 130 patients with anorexia nervosa at baseline (analysis by age corresponding to Table 1).** | | | | | | | | | | | | | | | |  |
| --- | --- | --- | --- | --- | --- | --- | --- | --- | --- | --- | --- | --- | --- | --- | --- | --- |
|  | PC-caloric avoidance factor score at T1 | | | | | | | | | | | | | | |  |
|  | Whole sample | | |  | | Adolescents | | | |  | | Adults | | | |  |
| Patients' characteristics | r | U | p | |  | | r | U | p | |  | | r | U | p | |
| Age | -0.106 |  | 0.229 | |  | | 0.173 |  | 0.212 | |  | | -0.121 |  | 0.297 | |
| Education |  | 1789 | 0.308 | |  | |  | 246 | 0.910 | |  | |  | 598 | 0.283 | |
| Working |  | 1003 | 0.837 | |  | |  | 35 | 0.442 | |  | |  | 546 | 0.905 | |
| Familial history of ED |  | 1432 | 0.187 | |  | |  | 227 | 0.506 | |  | |  | 515 | 0.273 | |
| Subtype |  | 2010 | 0.772 | |  | |  | 340 | 0.916 | |  | |  | 695 | 0.820 | |
| Age at onset | -0.105 |  | 0.234 | |  | | 0.197 |  | 0.153 | |  | | -0.166 |  | 0.152 | |
| Illness duration | -0.068 |  | 0.444 | |  | | -0.017 |  | 0.902 | |  | | -0.041 |  | 0.723 | |
| Current BMI | 0.008 |  | 0.925 | |  | | 0.094 |  | 0.498 | |  | | -0.032 |  | 0.787 | |
| Minimum lifetime BMI | 0.308 |  | **< .001** | |  | | 0.395 |  | **0.004** | |  | | 0.254 |  | **0.043** | |
| Maximum lifetime BMI | 0.120 |  | 0.201 | |  | | 0.273 |  | 0.052 | |  | | 0.052 |  | 0.686 | |
| BMI max-current | 0.104 |  | 0.270 | |  | | 0.209 |  | 0.141 | |  | | 0.054 |  | 0.672 | |
| BMI current-min | -0.286 |  | **0.002** | |  | | -0.286 |  | **0.042** | |  | | -0.289 |  | **0.021** | |
| BMI max-min | -0.037 |  | 0.696 | |  | | 0.076 |  | 0.598 | |  | | -0.088 |  | 0.489 | |
| Perceived BMI | 0.226 |  | **0.010** | |  | | 0.395 |  | **0.003** | |  | | 0.100 |  | 0.388 | |
| Perceptual distortion | 0.232 |  | **0.008** | |  | | 0.353 |  | **0.009** | |  | | 0.143 |  | 0.219 | |
| Subjective ideal BMI | -0.092 |  | 0.321 | |  | | 0.100 |  | 0.508 | |  | | -0.170 |  | 0.151 | |
| EAT Total | 0.520 |  | **< .001** | |  | | 0.596 |  | **< .001** | |  | | 0.464 |  | **< .001** | |
| EAT Dieting | 0.557 |  | **< .001** | |  | | 0.650 |  | **< .001** | |  | | 0.479 |  | **< .001** | |
| EAT Bulimia | 0.266 |  | **0.002** | |  | | 0.426 |  | **0.001** | |  | | 0.205 |  | 0.076 | |
| EAT Oral | 0.325 |  | **< .001** | |  | | 0.322 |  | **0.017** | |  | | 0.347 |  | **0.002** | |
| HADS anxiety score | 0.230 |  | **0.008** | |  | | 0.456 |  | **< .001** | |  | | 0.046 |  | 0.691 | |
| HADS anxiety syndrome |  | 653 | 0.128 | |  | |  | 75 | **0.006** | |  | |  | 218 | 0.680 | |
| HADS depression score | 0.100 |  | 0.258 | |  | | 0.279 |  | **0.041** | |  | | -0.036 |  | 0.758 | |
| HADS depression syndrome |  | 1536 | **0.037** | |  | |  | 206 | **0.043** | |  | |  | 612 | 0.371 | |
| PANAS positive | 0.005 |  | 0.953 | |  | | 0.070 |  | 0.617 | |  | | -0.008 |  | 0.948 | |
| PANAS negative | 0.211 |  | **0.016** | |  | | 0.359 |  | **0.008** | |  | | 0.098 |  | 0.400 | |
| WSAS | 0.165 |  | 0.061 | |  | | 0.301 |  | **0.027** | |  | | 0.070 |  | 0.545 | |
|  | PC-animal avoidance factor score at T1 | | | | | | | | | | | | | | |  |
|  | Whole sample | | |  | | Adolescents | | | |  | | Adults | | | |  |
| Patients' characteristics | r | U | p | |  | | r | U | p | |  | | r | U | p | |
| Age | -0.124 |  | 0.159 | |  | | 0.077 |  | 0.578 | |  | | -0.135 |  | 0.243 | |
| Education |  | 1633 | 0.077 | |  | |  | 233 | 0.704 | |  | |  | 558 | 0.134 | |
| Working |  | 1016 | 0.911 | |  | |  | 33 | 0.377 | |  | |  | 539 | 0.838 | |
| Familial history of ED |  | 1536 | 0.440 | |  | |  | 248 | 0.814 | |  | |  | 527 | 0.337 | |
| Subtype |  | 2064 | 0.972 | |  | |  | 287 | 0.298 | |  | |  | 664 | 0.583 | |
| Age at onset | -0.173 |  | **0.049** | |  | | -0.097 |  | 0.486 | |  | | -0.183 |  | 0.114 | |
| Illness duration | -0.053 |  | 0.546 | |  | | 0.183 |  | 0.184 | |  | | -0.048 |  | 0.683 | |
| Current BMI | -0.007 |  | 0.939 | |  | | 0.140 |  | 0.312 | |  | | -0.083 |  | 0.475 | |
| Minimum lifetime BMI | 0.251 |  | **0.007** | |  | | 0.338 |  | **0.015** | |  | | 0.198 |  | 0.116 | |
| Maximum lifetime BMI | 0.022 |  | 0.818 | |  | | 0.051 |  | 0.722 | |  | | 0.026 |  | 0.837 | |
| BMI max-current | 0.002 |  | 0.983 | |  | | -0.059 |  | 0.682 | |  | | 0.057 |  | 0.656 | |
| BMI current-min | -0.231 |  | **0.013** | |  | | -0.134 |  | 0.349 | |  | | -0.286 |  | **0.022** | |
| BMI max-min | -0.108 |  | 0.252 | |  | | -0.121 |  | 0.396 | |  | | -0.084 |  | 0.511 | |
| Perceived BMI | 0.263 |  | **0.003** | |  | | 0.481 |  | **< .001** | |  | | 0.106 |  | 0.362 | |
| Perceptual distortion | 0.275 |  | **0.002** | |  | | 0.401 |  | **0.003** | |  | | 0.185 |  | 0.109 | |
| Subjective ideal BMI | -0.253 |  | **0.006** | |  | | -0.163 |  | 0.280 | |  | | -0.283 |  | **0.015** | |
| EAT Total | 0.408 |  | **< .001** | |  | | 0.531 |  | **< .001** | |  | | 0.318 |  | **0.005** | |
| EAT Dieting | 0.441 |  | **< .001** | |  | | 0.599 |  | **< .001** | |  | | 0.315 |  | **0.006** | |
| EAT Bulimia | 0.191 |  | **0.029** | |  | | 0.343 |  | **0.011** | |  | | 0.134 |  | 0.249 | |
| EAT Oral | 0.260 |  | **0.003** | |  | | 0.272 |  | **0.047** | |  | | 0.272 |  | **0.017** | |
| HADS anxiety score | 0.285 |  | **0.001** | |  | | 0.398 |  | **0.003** | |  | | 0.196 |  | 0.090 | |
| HADS anxiety syndrome |  | 692 | 0.215 | |  | |  | 116 | **0.101** | |  | |  | 232 | 0.872 | |
| HADS depression score | 0.172 |  | 0.051 | |  | | 0.349 |  | **0.010** | |  | | 0.042 |  | 0.716 | |
| HADS depression syndrome |  | 1579 | 0.061 | |  | |  | 185 | **0.015** | |  | |  | 658 | 0.682 | |
| PANAS positive | -0.128 |  | 0.148 | |  | | -0.242 |  | 0.078 | |  | | -0.029 |  | 0.803 | |
| PANAS negative | 0.245 |  | **0.005** | |  | | 0.260 |  | 0.058 | |  | | 0.238 |  | **0.038** | |
| WSAS | 0.081 |  | 0.359 | |  | | 0.213 |  | 0.122 | |  | | -0.008 |  | 0.946 | |
|  | PC-vegetal avoidance factor score at T1 | | | | | | | | | | | | | | |  |
|  | Whole sample | | |  | | Adolescents | | | |  | | Adults | | | |  |
| Patients' characteristics | r | U | p | |  | | r | U | p | |  | | r | U | p | |
| Age | 0.089 |  | 0.316 | |  | | -0.191 |  | 0.167 | |  | | 0.124 |  | 0.285 | |
| Education |  | 1861 | 0.500 | |  | |  | 205 | 0.338 | |  | |  | 660 | 0.677 | |
| Working |  | 961 | 0.610 | |  | |  | 38 | 0.549 | |  | |  | 547 | 0.914 | |
| Familial history of ED |  | 1680 | 0.987 | |  | |  | 213 | 0.341 | |  | |  | 535 | 0.385 | |
| Subtype |  | 1516 | **0.009** | |  | |  | 282 | 0.259 | |  | |  | 480 | **0.013** | |
| Age at onset | -0.008 |  | 0.927 | |  | | -0.051 |  | 0.714 | |  | | -0.029 |  | 0.805 | |
| Illness duration | 0.105 |  | 0.233 | |  | | -0.154 |  | 0.268 | |  | | 0.144 |  | 0.215 | |
| Current BMI | 0.092 |  | 0.295 | |  | | 0.030 |  | 0.828 | |  | | 0.124 |  | 0.286 | |
| Minimum lifetime BMI | 0.195 |  | **0.037** | |  | | 0.071 |  | 0.620 | |  | | 0.294 |  | **0.018** | |
| Maximum lifetime BMI | 0.077 |  | 0.410 | |  | | 0.012 |  | 0.934 | |  | | 0.112 |  | 0.376 | |
| BMI max-current | 0.042 |  | 0.652 | |  | | 0.008 |  | 0.955 | |  | | 0.056 |  | 0.659 | |
| BMI current-min | -0.131 |  | 0.162 | |  | | -0.070 |  | 0.626 | |  | | -0.207 |  | 0.100 | |
| BMI max-min | -0.022 |  | 0.816 | |  | | -0.024 |  | 0.865 | |  | | -0.046 |  | 0.718 | |
| Perceived BMI | 0.028 |  | 0.753 | |  | | 0.010 |  | 0.941 | |  | | 0.043 |  | 0.713 | |
| Perceptual distortion | -0.047 |  | 0.594 | |  | | -0.013 |  | 0.928 | |  | | -0.062 |  | 0.593 | |
| Subjective ideal BMI | -0.053 |  | 0.565 | |  | | -0.267 |  | 0.072 | |  | | 0.038 |  | 0.753 | |
| EAT Total | 0.263 |  | **0.003** | |  | | 0.240 |  | 0.081 | |  | | 0.282 |  | **0.014** | |
| EAT Dieting | 0.230 |  | **0.008** | |  | | 0.229 |  | 0.096 | |  | | 0.249 |  | **0.030** | |
| EAT Bulimia | 0.190 |  | **0.031** | |  | | 0.089 |  | 0.521 | |  | | 0.239 |  | **0.038** | |
| EAT Oral | 0.221 |  | **0.011** | |  | | 0.263 |  | 0.054 | |  | | 0.183 |  | 0.113 | |
| HADS anxiety score | 0.213 |  | **0.015** | |  | | 0.141 |  | 0.308 | |  | | 0.276 |  | **0.016** | |
| HADS anxiety syndrome |  | 827 | 0.799 | |  | |  | 137 | 0.263 | |  | |  | 168 | 0.190 | |
| HADS depression score | 0.089 |  | 0.314 | |  | | 0.049 |  | 0.727 | |  | | 0.120 |  | 0.302 | |
| HADS depression syndrome |  | 1776 | 0.356 | |  | |  | 274 | 0.460 | |  | |  | 632 | 0.494 | |
| PANAS positive | -0.030 |  | 0.734 | |  | | -0.130 |  | 0.347 | |  | | 0.017 |  | 0.882 | |
| PANAS negative | 0.197 |  | **0.025** | |  | | 0.147 |  | 0.288 | |  | | 0.236 |  | **0.040** | |
| WSAS | 0.137 |  | 0.120 | |  | | 0.044 |  | 0.750 | |  | | 0.204 |  | 0.078 | |

| **Table S3.3. Predicting factors of food avoidance (analysis by age corresponding to Table 2).** | | | | | | | | | | | |
| --- | --- | --- | --- | --- | --- | --- | --- | --- | --- | --- | --- |
|  | PC-caloric avoidance factor score at T2 | | | | | | | | | | |
|  | Whole sample | | |  | Adolescents | | |  | Adults | | |
| Patients' characteristics at T1 | r | U | p |  | r | U | p |  | r | U | p |
| Age | -0.088 |  | 0.321 |  | -0.047 |  | 0.738 |  | -0.091 |  | 0.435 |
| Education |  | 1957 | 0.829 |  |  | 225 | 0.586 |  |  | 655 | 0.638 |
| Working |  | 1030 | 0.991 |  |  | 36 | 0.476 |  |  | 554 | 0.981 |
| Familial history of ED |  | 1622 | 0.748 |  |  | 233 | 0.588 |  |  | 597 | 0.869 |
| Subtype |  | 1773 | 0.160 |  |  | 299 | 0.408 |  |  | 595 | 0.205 |
| Age at onset | -0.263 |  | **0.003** |  | -0.193 |  | 0.161 |  | -0.321 |  | **0.005** |
| Illness duration | 0.034 |  | 0.700 |  | 0.149 |  | 0.281 |  | 0.069 |  | 0.554 |
| Current BMI | 0.014 |  | 0.879 |  | 0.172 |  | 0.212 |  | -0.089 |  | 0.446 |
| Minimum lifetime BMI | 0.144 |  | 0.126 |  | 0.268 |  | 0.057 |  | 0.06 |  | 0.64 |
| Maximum lifetime BMI | 0.159 |  | 0.089 |  | 0.192 |  | 0.177 |  | 0.17 |  | 0.179 |
| BMI max-current | 0.151 |  | 0.107 |  | 0.066 |  | 0.643 |  | 0.236 |  | 0.06 |
| BMI current-min | -0.118 |  | 0.209 |  | -0.019 |  | 0.897 |  | -0.177 |  | 0.161 |
| BMI max-min | 0.088 |  | 0.351 |  | 0.058 |  | 0.687 |  | 0.147 |  | 0.248 |
| Perceived BMI | 0.257 |  | **0.003** |  | 0.479 |  | **< .001** |  | 0.076 |  | 0.516 |
| Perceptual distortion | 0.241 |  | **0.006** |  | 0.374 |  | **0.005** |  | 0.138 |  | 0.233 |
| Subjective ideal BMI | -0.193 |  | 0.036 |  | -0.11 |  | 0.467 |  | -0.239 |  | **0.041** |
| EAT Total | 0.434 |  | **< .001** |  | 0.394 |  | **0.003** |  | 0.467 |  | **< .001** |
| EAT Dieting | 0.465 |  | **< .001** |  | 0.454 |  | **< .001** |  | 0.471 |  | **< .001** |
| EAT Bulimia | 0.242 |  | **0.006** |  | 0.259 |  | 0.059 |  | 0.253 |  | **0.027** |
| EAT Oral | 0.250 |  | **0.004** |  | 0.177 |  | 0.200 |  | 0.324 |  | **0.004** |
| HADS anxiety score | 0.281 |  | **0.001** |  | 0.309 |  | **0.023** |  | 0.255 |  | **0.026** |
| HADS anxiety syndrome |  | 622 | 0.080 |  |  | 118 | 0.112 |  |  | 206 | 0.53 |
| HADS depression score | 0.098 |  | 0.266 |  | 0.159 |  | 0.252 |  | 0.048 |  | 0.683 |
| HADS depression syndrome |  | 1605 | 0.080 |  |  | 244 | 0.194 |  |  | 589 | 0.255 |
| PANAS positive | -0.075 |  | 0.394 |  | -0.129 |  | 0.351 |  | -0.019 |  | 0.872 |
| PANAS negative | 0.276 |  | **0.001** |  | 0.187 |  | 0.177 |  | 0.356 |  | **0.002** |
| WSAS | 0.212 |  | **0.016** |  | 0.246 |  | 0.073 |  | 0.188 |  | 0.104 |
|  | PC-animal avoidance factor score at T2 | | | | | | | | | | |
|  | Whole sample | | |  | Adolescents | | |  | Adults | | |
| Patients' characteristics at T1 | r | U | p |  | r | U | p |  | r | U | p |
| Age | -0.097 |  | 0.273 |  | -0.065 |  | 0.639 |  | -0.091 |  | 0.437 |
| Education |  | 1924 | 0.708 |  |  | 224 | 0.572 |  |  |  |  |
| Working |  | 1011 | 0.882 |  |  | 32 | 0.347 |  |  |  |  |
| Familial history of ED |  | 1527 | 0.412 |  |  | 249 | 0.830 |  |  |  |  |
| Subtype |  | 1740 | 0.119 |  |  | 299 | 0.408 |  |  |  |  |
| Age at onset | -0.308 |  | **< .001** |  | -0.254 |  | 0.063 |  | -0.366 |  | **0.001** |
| Illness duration | 0.047 |  | 0.598 |  | 0.192 |  | 0.164 |  | 0.092 |  | 0.427 |
| Current BMI | -0.008 |  | 0.932 |  | 0.233 |  | 0.090 |  | -0.168 |  | 0.148 |
| Minimum lifetime BMI | 0.124 |  | 0.186 |  | 0.371 |  | **0.007** |  | -0.04 |  | 0.754 |
| Maximum lifetime BMI | -0.050 |  | 0.596 |  | -0.018 |  | 0.901 |  | -0.056 |  | 0.661 |
| BMI max-current | -0.070 |  | 0.456 |  | -0.191 |  | 0.179 |  | 0.028 |  | 0.823 |
| BMI current-min | -0.101 |  | 0.281 |  | -0.037 |  | 0.794 |  | -0.133 |  | 0.296 |
| BMI max-min | -0.115 |  | 0.222 |  | -0.209 |  | 0.142 |  | -0.037 |  | 0.773 |
| Perceived BMI | 0.209 |  | **0.017** |  | 0.483 |  | **< .001** |  | -0.02 |  | 0.865 |
| Perceptual distortion | 0.220 |  | **0.012** |  | 0.339 |  | **0.012** |  | 0.126 |  | 0.279 |
| Subjective ideal BMI | -0.243 |  | **0.008** |  | -0.189 |  | 0.207 |  | -0.279 |  | **0.017** |
| EAT Total | 0.263 |  | **0.002** |  | 0.349 |  | **0.010** |  | 0.189 |  | 0.102 |
| EAT Dieting | 0.282 |  | **0.001** |  | 0.406 |  | **0.002** |  | 0.163 |  | 0.159 |
| EAT Bulimia | 0.172 |  | 0.050 |  | 0.182 |  | 0.187 |  | 0.189 |  | 0.101 |
| EAT Oral | 0.130 |  | 0.142 |  | 0.183 |  | 0.185 |  | 0.1 |  | 0.391 |
| HADS anxiety score | 0.211 |  | **0.016** |  | 0.165 |  | 0.233 |  | 0.249 |  | **0.03** |
| HADS anxiety syndrome |  | 856 | 0.965 |  |  | 176 | 0.858 |  |  | 218 | 0.68 |
| HADS depression score | 0.141 |  | 0.109 |  | 0.2 |  | 0.147 |  | 0.091 |  | 0.432 |
| HADS depression syndrome |  | 1693 | 0.185 |  |  | 262 | 0.336 |  |  | 620 | 0.418 |
| PANAS positive | -0.217 |  | **0.013** |  | -0.372 |  | **0.006** |  | -0.085 |  | 0.465 |
| PANAS negative | 0.140 |  | 0.112 |  | 0.02 |  | 0.886 |  | 0.249 |  | **0.03** |
| WSAS | 0.095 |  | 0.282 |  | 0.141 |  | 0.309 |  | 0.062 |  | 0.593 |
|  | PC-vegetal avoidance factor score at T2 | | | | | | | | | | |
|  | Whole sample | | |  | Adolescents | | |  | Adults | | |
| Patients' characteristics at T1 | r | U | p |  | r | U | p |  | r | U | p |
| Age | 0.049 |  | 0.581 |  | -0.284 |  | 0.037 |  | 0.187 |  | 0.105 |
| Education |  | 1659 | 0.100 |  |  | 204 | 0.327 |  |  |  |  |
| Working |  | 911 | 0.383 |  |  | 18 | 0.077 |  |  |  |  |
| Familial history of ED |  | 1387 | 0.120 |  |  | 223 | 0.455 |  |  |  |  |
| Subtype |  | 1648 | 0.046 |  |  | 283 | 0.266 |  |  |  |  |
| Age at onset | -0.070 |  | 0.426 |  | -0.276 |  | **0.043** |  | 0.011 |  | 0.926 |
| Illness duration | 0.092 |  | 0.299 |  | -0.022 |  | 0.875 |  | 0.189 |  | 0.102 |
| Current BMI | 0.082 |  | 0.352 |  | 0.086 |  | 0.538 |  | 0.092 |  | 0.432 |
| Minimum lifetime BMI | 0.162 |  | 0.083 |  | 0.025 |  | 0.860 |  | 0.253 |  | **0.043** |
| Maximum lifetime BMI | -0.014 |  | 0.879 |  | -0.178 |  | 0.211 |  | 0.114 |  | 0.37 |
| BMI max-current | -0.052 |  | 0.579 |  | -0.226 |  | 0.110 |  | 0.085 |  | 0.506 |
| BMI current-min | -0.103 |  | 0.274 |  | 0.068 |  | 0.637 |  | -0.215 |  | 0.088 |
| BMI max-min | -0.098 |  | 0.295 |  | -0.195 |  | 0.171 |  | -0.022 |  | 0.865 |
| Perceived BMI | -0.009 |  | 0.915 |  | 0.077 |  | 0.580 |  | -0.085 |  | 0.465 |
| Perceptual distortion | -0.065 |  | 0.462 |  | 0.028 |  | 0.842 |  | -0.145 |  | 0.21 |
| Subjective ideal BMI | -0.005 |  | 0.955 |  | -0.147 |  | 0.329 |  | 0.04 |  | 0.735 |
| EAT Total | 0.091 |  | 0.303 |  | -0.075 |  | 0.590 |  | 0.237 |  | **0.039** |
| EAT Dieting | 0.084 |  | 0.342 |  | -0.032 |  | 0.819 |  | 0.177 |  | 0.127 |
| EAT Bulimia | 0.096 |  | 0.278 |  | -0.234 |  | 0.089 |  | 0.323 |  | **0.004** |
| EAT Oral | 0.040 |  | 0.652 |  | -0.011 |  | 0.936 |  | 0.095 |  | 0.416 |
| HADS anxiety score | -0.013 |  | 0.884 |  | -0.085 |  | 0.540 |  | 0.051 |  | 0.663 |
| HADS anxiety syndrome |  | 827 | 0.799 |  |  | 167 | 0.693 |  |  | 230 | 0.843 |
| HADS depression score | 0.119 |  | 0.179 |  | -0.013 |  | 0.924 |  | 0.236 |  | **0.04** |
| HADS depression syndrome |  | 1594 | 0.072 |  |  | 278 | 0.506 |  |  | 545 | 0.108 |
| PANAS positive | -0.146 |  | 0.098 |  | -0.209 |  | 0.129 |  | -0.085 |  | 0.467 |
| PANAS negative | -0.119 |  | 0.178 |  | -0.219 |  | 0.111 |  | -0.026 |  | 0.824 |
| WSAS | 0.042 |  | 0.635 |  | -0.123 |  | 0.374 |  | 0.192 |  | 0.097 |

| **Table S3.4. Clinical characteristics covarying with factor scores of food avoidance between two visits of 130 patients with anorexia nervosa (analysis by age corresponding to Table 3).** | | | | | | | | | | | | |
| --- | --- | --- | --- | --- | --- | --- | --- | --- | --- | --- | --- | --- |
|  | PC-caloric avoidance factor score difference (T2-T1) | | | | | | | | | | | |
|  | Whole sample | | |  | Adolescents | | |  | Adults | | | |
| Patients' characteristics (T2-T1) | r | U | p |  | r | U | p |  | r | U | p |  |
| Age | 0.028 |  | 0.754 |  | -0.25 |  | 0.068 |  | 0.043 |  | 0.712 |  |
| Current BMI | -0.227 |  | **0.009** |  | -0.292 |  | **0.032** |  | -0.192 |  | 0.097 |  |
| Perceived BMI | 0.075 |  | 0.396 |  | 0.073 |  | 0.601 |  | 0.084 |  | 0.472 |  |
| Perceptual distortion | 0.252 |  | **0.004** |  | 0.268 |  | **0.050** |  | 0.248 |  | **0.031** |  |
| EAT Total | 0.383 |  | **< .001** |  | 0.53 |  | **< .001** |  | 0.279 |  | **0.015** |  |
| EAT Dieting | 0.418 |  | **< .001** |  | 0.555 |  | **< .001** |  | 0.317 |  | **0.005** |  |
| EAT Bulimia | 0.255 |  | **0.003** |  | 0.313 |  | **0.021** |  | 0.233 |  | **0.043** |  |
| EAT Oral | 0.198 |  | **0.024** |  | 0.382 |  | **0.004** |  | 0.067 |  | 0.564 |  |
| HADS anxiety score | 0.161 |  | 0.067 |  | 0.427 |  | **0.001** |  | -0.025 |  | 0.828 |  |
| Anxiety remission |  | 826 | 0.078 |  |  | 128 | 0.180 |  |  | 302 | 0.246 |  |
| HADS depression score | 0.182 |  | **0.039** |  | 0.351 |  | **0.009** |  | 0.08 |  | 0.494 |  |
| Depression remission |  | 1150 | **0.011** |  |  | 125 | **0.003** |  |  | 499 | 0.365 |  |
| PANAS positive | -0.042 |  | 0.633 |  | -0.101 |  | 0.470 |  | 0 |  | 0.997 |  |
| PANAS negative | 0.131 |  | 0.137 |  | 0.287 |  | **0.035** |  | 0.042 |  | 0.718 |  |
| WSAS | 0.071 |  | 0.424 |  | 0.179 |  | 0.194 |  | 0.002 |  | 0.987 |  |
| Delay between visits | 0.024 |  | 0.784 |  | -0.057 |  | 0.682 |  | 0.154 |  | 0.183 |  |
|  | PC-animal avoidance factor score difference (T2-T1) | | | | | | | | | | | |
|  | Whole sample | | |  | Adolescents | | |  | Adults | | | |
| Patients' characteristics | r | U | p |  | r | U | p |  | r | U | p |  |
| Age | 0.031 |  | 0.727 |  | -0.181 |  | 0.190 |  | 0.058 |  | 0.618 |  |
| Current BMI | -0.071 |  | 0.419 |  | 0.049 |  | 0.726 |  | -0.136 |  | 0.243 |  |
| Perceived BMI | 0.019 |  | 0.827 |  | -0.066 |  | 0.636 |  | 0.079 |  | 0.500 |  |
| Perceptual distortion | 0.065 |  | 0.466 |  | -0.112 |  | 0.421 |  | 0.186 |  | 0.107 |  |
| EAT Total | 0.324 |  | **< .001** |  | 0.266 |  | 0.051 |  | 0.377 |  | **< .001** |  |
| EAT Dieting | 0.351 |  | **< .001** |  | 0.33 |  | **0.015** |  | 0.374 |  | **< .001** |  |
| EAT Bulimia | 0.230 |  | **0.008** |  | 0.265 |  | 0.053 |  | 0.217 |  | 0.060 |  |
| EAT Oral | 0.161 |  | 0.068 |  | 0.025 |  | 0.858 |  | 0.265 |  | **0.021** |  |
| HADS anxiety score | 0.155 |  | 0.079 |  | 0.235 |  | 0.087 |  | 0.098 |  | 0.401 |  |
| Anxiety remission |  | 910 | 0.221 |  |  | 159 | 0.558 |  |  | 296 | 0.213 |  |
| HADS depression score | 0.103 |  | 0.245 |  | 0.14 |  | 0.311 |  | 0.08 |  | 0.492 |  |
| Depression remission |  | 1334 | 0.115 |  |  | 156 | **0.025** |  |  | 538 | 0.651 |  |
| PANAS positive | -0.073 |  | 0.410 |  | 0.111 |  | 0.424 |  | -0.182 |  | 0.115 |  |
| PANAS negative | 0.200 |  | **0.022** |  | 0.093 |  | 0.504 |  | 0.263 |  | **0.022** |  |
| WSAS | 0.119 |  | 0.176 |  | -0.117 |  | 0.401 |  | 0.262 |  | **0.022** |  |
| Delay between visits | 0.072 |  | 0.417 |  | -0.058 |  | 0.677 |  | 0.184 |  | 0.111 |  |
|  | PC-vegetal avoidance factor score difference (T2-T1) | | | | | | | | | | | |
|  | Whole sample | | |  | Adolescents | | |  | Adults | | | |
| Patients' characteristics | r | U | p |  | r | U | p |  | r | U | p |  |
| Age | -0.034 |  | 0.700 |  | -0.114 |  | 0.411 |  | 0.054 |  | 0.645 |  |
| Current BMI | 0.172 |  | 0.050 |  | 0.121 |  | 0.384 |  | 0.2 |  | 0.083 |  |
| Perceived BMI | 0.035 |  | 0.691 |  | -0.044 |  | 0.751 |  | 0.077 |  | 0.506 |  |
| Perceptual distortion | -0.100 |  | 0.257 |  | -0.124 |  | 0.372 |  | -0.094 |  | 0.422 |  |
| EAT Total | 0.125 |  | 0.157 |  | 0.165 |  | 0.234 |  | 0.085 |  | 0.466 |  |
| EAT Dieting | 0.088 |  | 0.320 |  | 0.148 |  | 0.285 |  | 0.038 |  | 0.744 |  |
| EAT Bulimia | 0.119 |  | 0.177 |  | 0.177 |  | 0.200 |  | 0.058 |  | 0.619 |  |
| EAT Oral | 0.118 |  | 0.181 |  | 0.104 |  | 0.455 |  | 0.127 |  | 0.273 |  |
| HADS anxiety score | 0.043 |  | 0.629 |  | -0.081 |  | 0.561 |  | 0.138 |  | 0.236 |  |
| Anxiety remission |  | 1084 | 0.920 |  |  | 161 | 0.590 |  |  | 367 | 0.814 |  |
| HADS depression score | -0.044 |  | 0.618 |  | 0.043 |  | 0.759 |  | -0.111 |  | 0.340 |  |
| Depression remission |  | 1588 | 0.818 |  |  | 252 | 0.780 |  |  | 525 | 0.546 |  |
| PANAS positive | -0.078 |  | 0.381 |  | 0.014 |  | 0.921 |  | -0.165 |  | 0.154 |  |
| PANAS negative | 0.146 |  | 0.098 |  | 0.006 |  | 0.966 |  | 0.241 |  | **0.036** |  |
| WSAS | 0.099 |  | 0.262 |  | 0.033 |  | 0.814 |  | 0.158 |  | 0.173 |  |
| Delay between visits | -0.059 |  | 0.509 |  | 0.026 |  | 0.854 |  | -0.027 |  | 0.820 |  |

| **Table S3.5. Characteristics of 130 patients with anorexia nervosa who reintroduced at least one fat food (versus did not) after four months of treatment (analysis by age corresponding to Table 4).** | | | | | | | | | | | |
| --- | --- | --- | --- | --- | --- | --- | --- | --- | --- | --- | --- |
|  | Whole sample | | |  | Adolescents | | |  | Adults | | |
|  | ꭓ2 | U | p |  | ꭓ2 | U | p |  | ꭓ2 | U | p |
| Age |  | 1604 | 0.386 |  |  | 275 | 0.368 |  |  | 573 | 0.958 |
| Education (high) | 2.11 |  | 0.146 |  | 0.48 |  | 0.487 |  | 1.29 |  | 0.257 |
| Working (presently) | 0.01 |  | 0.903 |  | 1.26 |  | 0.262 |  | 0.35 |  | 0.552 |
| Familial history of ED (yes) | 0.93 |  | 0.336 |  | 2.65 |  | 0.104 |  | 0.02 |  | 0.877 |
| Subtype (restrictive) | 3.00 |  | 0.083 |  | 0.35 |  | 0.554 |  | 3.57 |  | 0.059 |
| Age at onset |  | 1726 | 0.807 |  |  | 310 | 0.803 |  |  | 473 | 0.223 |
| Illness duration |  | 1510 | 0.178 |  |  | 259 | 0.220 |  |  | 483 | 0.274 |
| BMI |  |  |  |  |  |  |  |  |  |  |  |
| At T1 |  | 1609 | 0.402 |  |  | 264 | 0.271 |  |  | 391 | **0.030** |
| Difference |  | 1707 | 0.734 |  |  | 300 | 0.660 |  |  | 573 | 0.958 |
| Minimum lifetime BMI |  | 1325 | 0.559 |  |  | 203 | 0.088 |  |  | 399 | 0.681 |
| Maximum lifetime BMI |  | 1345 | 0.642 |  |  | 205 | 0.095 |  |  | 353 | 0.274 |
| BMI max-current |  | 1274 | 0.372 |  |  | 254 | 0.494 |  |  | 419 | 0.901 |
| BMI current-min |  | 1322 | 0.548 |  |  | 251 | 0.454 |  |  | 319 | 0.113 |
| BMI max-min |  | 1308 | 0.494 |  |  | 246 | 0.390 |  |  | 387 | 0.552 |
| Perceived BMI |  |  |  |  |  |  |  |  |  |  |  |
| At T1 |  | 1734 | 0.838 |  |  | 287 | 0.494 |  |  | 551 | 0.760 |
| Difference |  | 1571 | 0.298 |  |  | 255 | 0.206 |  |  | 576 | 0.986 |
| Perceptual distortion |  |  |  |  |  |  |  |  |  |  |  |
| At T1 |  | 1556 | 0.268 |  |  | 308 | 0.769 |  |  | 421 | 0.070 |
| Difference |  | 1474 | 0.127 |  |  | 286 | 0.491 |  |  | 453 | 0.148 |
| Subjective ideal BMI |  | 1341 | 0.377 |  |  | 221 | 0.661 |  |  | 386 | 0.075 |
| EAT total |  |  |  |  |  |  |  |  |  |  |  |
| At T1 |  | 1383 | 0.047 |  |  | 243 | 0.139 |  |  | 457 | 0.163 |
| Difference |  | 1288 | 0.013 |  |  | 228 | 0.078 |  |  | 431 | 0.088 |
| EAT dieting |  |  |  |  |  |  |  |  |  |  |  |
| At T1 |  | 1413 | 0.066 |  |  | 227 | 0.076 |  |  | 488 | 0.301 |
| Difference |  | 1370 | 0.040 |  |  | 251 | 0.180 |  |  | 457 | 0.162 |
| EAT bulimia |  |  |  |  |  |  |  |  |  |  |  |
| At T1 |  | 1222 | **0.005** |  |  | 224 | 0.066 |  |  | 406 | 0.046 |
| Difference |  | 1497 | 0.155 |  |  | 282 | 0.442 |  |  | 449 | 0.134 |
| EAT oral |  |  |  |  |  |  |  |  |  |  |  |
| At T1 |  | 1693 | 0.678 |  |  | 315 | 0.876 |  |  | 546 | 0.718 |
| Difference |  | 1215 | **0.004** |  |  | 190 | **0.013** |  |  | 438 | 0.104 |
| HADS anxiety |  |  |  |  |  |  |  |  |  |  |  |
| At T1 |  | 1243 | **0.007** |  |  | 220 | 0.057 |  |  | 415 | 0.058 |
| Remission | 4.50 |  | 0.034 |  | 3.60 |  | 0.058 |  | 1.40 |  | 0.236 |
| HADS depression |  |  |  |  |  |  |  |  |  |  |  |
| At T1 |  | 1548 | 0.249 |  |  | 287 | 0.501 |  |  | 505 | 0.397 |
| Remission | 0.12 |  | 0.728 |  | 1.27 |  | 0.260 |  | 0.21 |  | 0.645 |
| PANAS positive |  |  |  |  |  |  |  |  |  |  |  |
| At T1 |  | 1709 | 0.741 |  |  | 311 | 0.818 |  |  | 492 | 0.320 |
| Difference |  | 1489 | 0.147 |  |  | 230 | 0.084 |  |  | 537 | 0.642 |
| PANAS negative |  |  |  |  |  |  |  |  |  |  |  |
| At T1 |  | 1360 | 0.035 |  |  | 240 | 0.123 |  |  | 452 | 0.144 |
| Difference |  | 1542 | 0.238 |  |  | 223 | 0.064 |  |  | 570 | 0.930 |
| WSAS |  |  |  |  |  |  |  |  |  |  |  |
| At T1 |  | 1408 | 0.062 |  |  | 248 | 0.165 |  |  | 473 | 0.224 |
| Difference |  | 1711 | 0.747 |  |  | 304 | 0.713 |  |  | 558 | 0.825 |
| Delay between visits |  | 1719 | 0.778 |  |  | 270 | 0.326 |  |  | 536 | 0.634 |
| *BMI: body mass index; EAT: eating attitudes test-26; ED: eating disorder; HADS: hospital anxiety and depressive scale; p: p-value; PANAS: positive and negative affect schedule; SD: standard deviation; T1: at baseline; U: Mann-Whitney U; WSAS: work and social adjustment scale; ꭓ2: chi-squared test. Bold numbers indicate p-values<0.05.* | | | | | | | | | | | |
